# Predicting Ambulance Transport for Heat-Related Illness in Working Populations Under Climate Change and Evaluating Preventive Behaviors as Adaptation Policies in Japan

**DOI:** 10.3390/epidemiologia7030060

**Published:** 2026-05-04

**Authors:** Shintaro Yurugi, Hiroshi Nishiura

**Affiliations:** Center for Health Security, Graduate School of Medicine, Kyoto University, Yoshidakonoecho, Sakyoku, Kyoto 606-8501, Japan

**Keywords:** emergency transport, heatstroke, risk reduction, climate change, statistical model

## Abstract

Background/Objectives: Since June 2025, Japan has mandated countermeasures to prevent outdoor laborers from developing heat-related illness at work. However, the extent to which preventive behaviors can reduce the actual heatstroke risk has not been quantified. The present study aimed to (i) project future trends in the daily number of heat-related ambulance transports in the working population under climate change, and (ii) evaluate the population-level preventive impact of workplace-adopted preventive behaviors using effect estimates from observational data. Methods: Using daily maximum wet-bulb globe temperature, a long-term future projection of heat-related ambulance transports was performed in the working population. A cross-sectional survey was carried out to infer the effect size of behavioral interventions. The effectiveness of taking preventive behaviors was evaluated by increasing the coverage rate of workers adhering to all four behaviors (current: 23%): (i) regular hydration, (ii) use of an air-cooling vest, (iii) checking their own health condition before work, and (iv) recognizing warning signs. Theoretical scenarios in which workplace instructions to workers or teams increased adherence by 50%, 100%, and 300% relative to baseline were considered, corresponding to coverage rates at 34%, 45%, and 91%, respectively, and we evaluated the associated reduction in heatstroke risk. Results: Many future years were projected to have higher annual levels of heat-related ambulance transports than the median value from 2018–2024, indicating a long-term increasing trend. Even when all four possible countermeasures were implemented at an additional 50%, 100% or 300% from the current rate, the expected relative risk reduction in transports was 3.2%, 6.3%, and 19.0%, respectively, indicating only a small effect on future projected heat-related illnesses. Conclusions: The number of heat-related ambulance transports is expected to increase; however, the relative risk reduction with behavioral intervention is likely limited. A fundamental overhaul of working regulations and environment (e.g., drastic shift in working hours to earlier morning) is required via adaptation policies, and mitigation of climate change is vital.

## 1. Introduction

Heatstroke is a severe form of heat-related illness characterized by lethal pathophysiology involving hyperthermia and central nervous system dysfunction [1]. Causatively, the disease is classified into classic heatstroke, mainly attributable to environmental exposure such as a heat wave, and exertional heatstroke triggered by physical activity and exertion [2]. In recent decades, heat exposure has become recognized as a global public health problem along with the issue of climate change. The health impact of and possible countermeasures against heat exposure should be systematically investigated [3]. Heat exposure not only raises the risk of health hazards and workplace productivity decline; exposure to extreme heat also elevates the demand for emergency medical support [4].

The health impacts of climate change have been quantitatively evaluated using empirical data, and additional heat exposure attributable to human-induced exacerbation of climate change is reported to increase the burden of heat-related mortality [5]. Accordingly, as part of countermeasures against extreme heat, heat-related health warning systems and action plans have been formulated worldwide, including implementation of such systems in France [6], evaluation of changes in mortality before and after countermeasures against extreme heat [7], and critical assessment of heat-related interventions [8]. As a practical metric of heat exposure, the wet-bulb globe temperature (WBGT) is recognized globally, and its strengths and limitations have been discussed [9]. In Japan, the association between the daily maximum temperature or WBGT and ambulance transports for patients with heatstroke has been shown to vary with age and geographic location [10]. Using climate change scenarios, the long-term future baseline of heat-related ambulance transports has been studied to estimate the future health burden [11].

Published research on heat-related illness has tended to focus on vulnerable age groups, such as young children, or epidemiological evaluations of adaptation policies to reduce heat-related illness among older people [12]. However, working adults aged 18–64 years constitute a substantial proportion of heat-related ambulance transports [10]. Although occupational exposure limits for heat stress have been discussed [13], possible intervention policies focusing on working populations have yet to be extensively evaluated. In Japan, the Ordinance on Industrial Safety and Health was revised on 1 June 2025, mandating that business operators establish an intervention team, prepare action plans, and ensure adequate communication in response to certain environmental conditions (e.g., continuous work for more than 1 h with WBGT greater than 28 °C or 31 °C or a total daily working time of more than 4 h under conditions of heat exposure) [14]. Although the actual preventive measures depend on each individual business operator, most currently adopted interventions are aimed at changing behaviors during working hours. Although the mandated countermeasures are in place, how efficiently these measures reduce heat-related illness in the working population under climate change is not yet fully understood.

The purpose of the present study was to predict future increases in heat-related ambulance transports among the working population and to evaluate possible adaptation policies to reduce the related disease burden.

## 2. Materials and Methods

In the present study, we combined two epidemiological approaches: (i) statistical modeling to project future heat-related illness using WBGT values, and (ii) estimating the effect size of adaptation measures via a cross-sectional epidemiological survey.

### 2.1. Epidemiological Dataset

For the exposition of our evaluation method, the present study focused on Shiga Prefecture, located east of Kyoto and roughly at the center of Japan, with a population of 1.4 million. Shiga Prefecture is surrounded by mountains over 1000 m high, and one-sixth of its land area is occupied by Lake Biwa—the largest lake in the country—at the center of the prefecture.

#### 2.1.1. Datasets for the Prediction Model

To create the baseline of ambulance transports related to heat-related illness cases, we used three pieces of data: (i) daily ambulance transports of patients with heatstroke, (ii) the daily maximum WBGT, and (iii) observed meteorological data from the local weather observatory station. For (i), the daily count among patients aged 18–64 years was obtained from local ambulance dispatch stations, spanning from May to September in each year [15]. For both (ii) and (iii), we retrieved observation data from 2018 to 2024 to establish the meteorological baseline at Otsu observatory station [16,17]. The WBGT values were also directly observed for the baseline years.

#### 2.1.2. Climate Scenario Data

We used future meteorological data from climate change scenarios of the Coupled Model Intercomparison Project Phase 6 (CMIP6), a project of the World Climate Research Programme. Specifically, the National Institute of Environmental Studies openly shared three climate change scenarios or models [18]: (i) the Model for Interdisciplinary Research on Climate 6 (MIROC6) [19], (ii) Meteorological Research Institute Earth System Model (MRI-ESM-2.0) [20], and (iii) Institut Pierre-Simon Laplace climate model (IPSL-CM6A-LR) [21]. All of these models included a carbon-neutral scenario (e.g., representative concentration pathway [RCP] 1.9) in addition to other possible scenarios. Specifying the geographic coordinates of Shiga Prefecture, we retrieved meteorological variables up to the year 2100 for RCP1.9, RCP2.6, RCP4.5, and RCP8.5. Predicted values of WBGT in the future were computed using an approximation formula developed by Ono and Tonouchi [22]. That is, given the maximum daily temperature (°C) *T*_asmax_, relative humidity (%) *RH*, global solar radiation (kW/m^2^) *SR* measured using a horizontally installed solar radiation meter, and the average wind speed (m/s) *WS*, the daily maximum WBGT *T* was calculated as follows:

(1) $$T=0.735T_{asmax}+0.0374RH+0.00292T_{asmax}RH+7.619SR-4.557{SR}^{2}-0.0572WS-4.064$$

We evaluated the risk of ambulance transport due to heat-related illness at the population level; therefore, we also collected estimated population size data from 2018 to 2024 [23] and future population projections from an information platform of climate change adaptation in Japan [24].

#### 2.1.3. Cross-Sectional Epidemiological Survey

To understand the effect size of possible intervention measures, we carried out a cross-sectional study among Japanese individuals aged 18–64 years. Participants were recruited from registered monitors of a private internet survey company, Mellinks Ltd. (Nerima-ku, Tokyo, Japan), encompassing a total of more than three million registered users. Sampling was carried out via convenience sampling, using a first-come first-served principle (i.e., advertising for respondents, participants voluntarily self-enrolling in the survey, and closing recruitment once the required sample size was reached). Respondents did not receive any financial remuneration; however, by completing the questionnaire, they received company points that could later be redeemed in exchange for certain items. The survey took place from 22 to 27 February 2024, with participants completing the questionnaire online. The survey was designed following our published study [23], querying experiences of heat-related illness and also implementation of protective countermeasures. Because the frequency of heat-related illness is rare among workers, we performed 1:2 sampling of respondents with and without heatstroke, following our published study [25]. Considering a reported proportion of older people who regularly drink water of 77% [25], we assumed that at least 70% of workers who had experienced an episode of heat-related illness regularly drank water. Moreover, assuming that 6.5% or more of respondents had not experienced a heat-related episode and considering 15 respondents did not return a valid survey, with an α error of 0.05 and 1 − β of 0.80, the total sample size was determined to be 1650. Interventions included drinking water, taking regular rests to cool down, checking one’s health condition in advance of work, and using special clothing equipped with air circulation (i.e., an air-cooling vest). Information about interventions as well as the presence of underlying medical conditions was collected; these included diabetes, hypertension, heart disease, kidney disease, Parkinson’s disease, epilepsy, and depression, conditions that are documented as being associated with the risk of heatstroke. The outcome of questionnaire survey was the presence of at least one heat-related illness episode. Preventive behaviors were assessed using dichotomized yes/no questions, and responses were coded as binary variables including (i) regular hydration (drinking water at regular intervals during work), (ii) use of an air-cooling vest, (iii) checking one’s health condition before work, and (iv) recognition of warning alerts (e.g., national/local heat-illness warning alerts). We also collected information on sociodemographic variables, the working environment, and underlying comorbidities.

### 2.2. Statistical Analysis

#### 2.2.1. Building a Prediction Model of Ambulance Transport Using the WBGT

For the sake of prediction, we exploited the relationship between ambulance transports per 100,000 workers and the daily maximum WBGT value. The statistical model assumed that the risk of heat-related transport increased exponentially with an elevation in the WBGT above a certain threshold level. Letting *λ* be the coefficient of exponential growth of risk, *T*_w_ the threshold WBGT level, and *N* the baseline risk of heat-related illness even below the threshold, we calculated the expected daily risk of ambulance transport per 100,000 given the WBGT on day *d*, *T*_d_ as

(2) $$E(n\left(T_{d}\right))=\left\{\begin{array}{ll} N & ,\;\mathrm{for}\;T_{d}<T_{w}\\ Nexp\left(\lambda \left(T_{d}-T_{w}\right)\right) & ,\;\mathrm{for}{\;T}_{w}\leq T_{d} \end{array}\right.$$

To quantify the model, we used the baseline years from 2018 to 2024. Parameter estimation was carried out using the maximum likelihood method. We assumed that the daily number of ambulance transports followed a Poisson distribution, with the expected number informed by Equation (2), and that the negative logarithm of the likelihood was minimized.

#### 2.2.2. Future Projection of Ambulance Transports

We used climate change scenarios to compute the projected number of heat-related ambulance transports in the future from 2025 until 2100. Equation (1) was used to compute the daily maximum WBGT for each RCP; subsequently, the parameterized model (2) was used to calculate the expected number of ambulance transports. All climate change scenarios using MIROC6, MRI-ESM-2.0, and IPSL-CM6A-LR were examined.

To compute the 95% confidence intervals (CIs) of the projected number of ambulance transports, we used a parametric bootstrap method. We first used the variance matrix from maximum likelihood estimation, and resampled parameters with a multivariate normal distribution. To further address the observation uncertainty, Poisson variations were allowed for the projected number from Equation (2). After performing a total of 5000 resampling experiments, the resulting 2.5th and 97.5th percentiles of projected ambulance transports were taken as the 95% CI.

#### 2.2.3. Evaluating Adaptation Strategies or Policies

We first evaluated preventive measures taken from May to September 2023, as reported in our cross-sectional survey. The outcome (i.e., heat-related illness episodes) as well as all interventions were dichotomized, and statistical associations between each intervention and heat-related illness were examined. We first explored the univariate associations, computing odds ratios (ORs) in logistic regression. Subsequently, including interventions that were potentially practicalized and significant in univariate analysis, we applied a multivariate logistic model adjusted for potential confounders. The adjusted variables included age, sex, the presence of children, working environment, and underlying comorbidities. The multivariate model as well as more causal approaches were applied. Propensity score matching was performed using the adjusted variables, with a propensity score standard deviation of 0.2 via nearest neighbor matching without replacement. Using the same propensity score, we also computed the effect size of interventions using the inverse probability of treatment weighting method.

We subsequently assessed the population impact of adaptation measures using the predicted risk of heat-related illness in an individual *i*, i.e.,

(3) $$ln\left(\frac{y_{i}}{1-y_{i}}\right)=\sum_{j}\beta_{j}x_{ij}+\sum_{j,k}\delta_{jk}x_{ij}x_{ik}+\gamma$$

where $\beta_{j}$ and $\delta_{jk}$ represent the coefficient of intervention variable *j* and interaction between *j* and *k*, respectively, and $x_{ij}$ is the dichotomous variable representing the implementation of intervention *j* by individual *i*. Parameter γ is a constant. The best model was selected by comparing Akaike information criterion values, accounting for all possible combinations of interaction terms. To evaluate behavioral adaptation measures, we assumed that all four interventions are commonly implemented. Let *p* and *q* be the baseline risk without interventions and the risk with all four interventions, respectively. The OR of the combined four measures compared with the absence of interventions is calculated as

(4) $$\begin{array}{c} ln\left(\frac{p}{1-p}\right)=\hat{\gamma ,}\\ ln\left(\frac{q}{1-q}\right)=\sum_{j}{\hat{\beta }}_{j}+\sum_{j,k}{\hat{\delta }}_{jk}+\hat{\gamma ,} \end{array}$$

leading to

(5) $$exp\left(\sum_{j}{\hat{\beta }}_{j}+\sum_{j,k}{\hat{\delta }}_{jk}\right)=\frac{\frac{q}{1-q}}{\frac{p}{1-p}}.$$

We used the population attributable fraction *f*(*w*) to evaluate the impact of combined interventions with the coverage rate 1 − *w*. In the observed data, a certain fraction of people performed all four measures. Let *i*_r_(*t*) and *i*_0_(*t*) be the rate of ambulance transports due to heat-related illness in reality and also in the absence of interventions. With the actual implementation rate (*w*) of interventions,

(6) $$f\left(w\right)=\frac{i_{r}\left(t\right)-i_{0}\left(t\right)}{i_{r}\left(t\right)}.$$

It should be noted that *f*(*w*) is also computed using *w* and the risk ratio of interventions. When *w* was varied to *w*′ owing to interventions, an equation similar to (6) is obtained. Cancelling *i*_0_(*t*) using two equations, we then obtained the rate of ambulance transports under interventions *i*_v_(*t*) as

(7) $$i_{v}\left(t\right)=i_{r}\left(t\right)\frac{1-f(w)}{1-f(w^{\prime })};$$

thus, the survey-based effect size and the proportion (coverage rate) implementing interventions could inform *i*_v_(*t*). When additional intervention policies are in place, the corresponding proportion is increased by *k* times. Theoretically, we explored the implementation coverage of a 50%, 100%, or 300% increase (i.e., *k* = 1.5, 2.0, or 4.0).

## 3. Results

### 3.1. Projection of Heat-Related Illness

**Figure 1:**
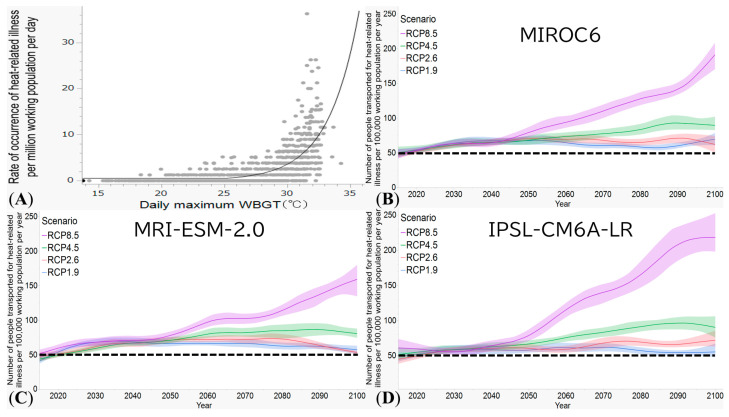
Risk of heat-related ambulance transport as a function of wet-bulb globe temperature and long-term projection until 2100. (**A**) Risk of heat-related ambulance transport as a function of wet-bulb globe temperature (WBGT) (2018–2024, Shiga Prefecture): The exponential model (black line) indicates the relationship between daily maximum WBGT and the daily number of heat-related ambulance transports. The vertical axis is the number of cases per million working people per day; the horizontal axis is the daily maximum WBGT. Gray circles represent observed values, and the solid line shows the solution of the exponential model. (**B**–**D**) Projected number of heatstroke-related ambulance transports among the working population in Shiga Prefecture from 2015 to 2100 using three climate change models MIROC6, MRI-ESM-2.0, and IPSL-CM6A-LR, respectively, for panels (**B**–**D**). The vertical axis is the number of heatstroke-related ambulance transports per 100,000 workers; the horizontal axis represents the calendar year. Lines represent smoothed projections (5-year moving average). The horizontal dotted line in each panel indicates the median value of the baseline period for the number of ambulance transports due to heat-related illness. MIROC6, Model for Interdisciplinary Research on Climate 6; MRI-ESM-2.0, Meteorological Research Institute Earth System Model; IPSL-CM6A-LR, Institut Pierre-Simon Laplace climate model.

Using observed data for ambulance transports from 2018 to 2024, we estimated the threshold WBGT to be *T*_w_ = 25.3 (95% CI: 24.7, 25.8), *λ* = 0.41 (95% CI: 0.38, 0.44), and *N* = 0.51 (95% CI: 0.42, 0.60). Figure 1A shows a comparison between the observed and predicted number of heat-related ambulance transports as a function of the daily maximum WBGT. Using the parameterized statistical model, Figure 1B–D shows the projection results using MIROC6, MRI-ESM-2.0, and IPSL-CM6-LR, respectively, from 2025 to 2100 for each RCP. Compared with the median value from 2018 to 2024, the number of ambulance transports per 100,000 workers was expected to increase in the long term for each RCP, including the carbon-neutral scenario. Under the worst scenario (RCP8.5), MIROC6, MRI-ESM-2.0, and IPSL-CM6-LR yielded an additional relative increase of 36%, 31%, and 58% in the daily number of ambulance transports in 2050, respectively.

### 3.2. Association Between Preventive Behaviors and Risk of Heat-Related Illness

A total of 1650 working-age respondents participated in the cross-sectional survey, consisting of 550 individuals who had experienced at least one heat-related illness episode and 1100 individuals without such an episode. Male respondents accounted for 825 participants (50.0%), and the mean age (standard deviation) was 45.5 (15.0) years. Among participants, 49.2% reported taking breaks during working hours, and 74.6% reported regular hydration. Regarding underlying medical conditions, 90 (5.5%) had diabetes mellitus, 196 (11.9%) had hypertension, and 66 (4.0%) had depression. Overall, 1311 (79.5%) reported no comorbidities. Table 1 summarizes the comparison between participants with and without a heat-related illness episode. Participants with an episode tended to have a higher proportion of workers with children and exercise habits. By contrast, participants without an episode tended to have higher proportions of those who recognized warning alerts, used an air-cooling vest, practiced regular hydration, and checked their health condition before work.

Table 2 shows the results of univariate analysis for the risk of heat-related illness episodes in association with the dependent variables. Among feasible preventive behaviors, regular hydration, use of an air-cooling vest, recognition of warning alerts, and workers checking their health condition before work were inversely associated with the outcome, with crude ORs of 0.6 (95% CI: 0.5, 0.7), 0.6 (95% CI: 0.5, 0.8), 0.8 (95% CI: 0.7, 1.0), and 0.8 (95% CI: 0.6, 1.0), respectively. The crude OR for being aware of the need for prevention was 0.9 (95% CI: 0.7, 1.1).

To obtain effect sizes of feasible preventive behaviors as adaptation policies, we examined the adjusted ORs for the four interventions described above (Table 3). Even after adjustment for covariates, estimated adjusted ORs were similar in magnitude to the crude ORs, suggesting that each of the four preventive behaviors may have a protective effect. Because practical workplace countermeasures are likely to be implemented together, we additionally considered the combined implementation of all four behaviors. The OR of having a heat-related illness episode among individuals implementing all four behaviors compared with those not implementing all four measures was 0.7 (95% CI: 0.6, 0.9). The proportion implementing all four interventions was 23% (77% did not carry out at least one preventive behavior).

### 3.3. Relative Risk Reduction in Heat-Related Illness at the Population Level by Elevating the Coverage Rate of Preventive Behaviors

Table 4 summarizes the population-level risk reduction under adaptation policies comprising four preventive behaviors. When the implementation coverage rate was theoretically increased by 50% (coverage 34%), the relative risk reduction in heat-related illness ambulance transports was 3.2% (95% CI: 0.6, 5.4). When the implementation rate increased by 100% (coverage 45%), the relative risk reduction was 6.3% (95% CI: 1.2, 10.8). Under the most intensive scenario (a 300% increase; coverage 91%), the relative risk reduction was 19.0% (95% CI: 3.5, 32.3). The corresponding population attributable fraction (PAFs) for each coverage was 19.0% (95% CI: 3.4, 32.9), 16.3% (95% CI: 2.8, 28.9), and 3.1% (95% CI: 0.5, 6.3), respectively.

**Figure 2:**
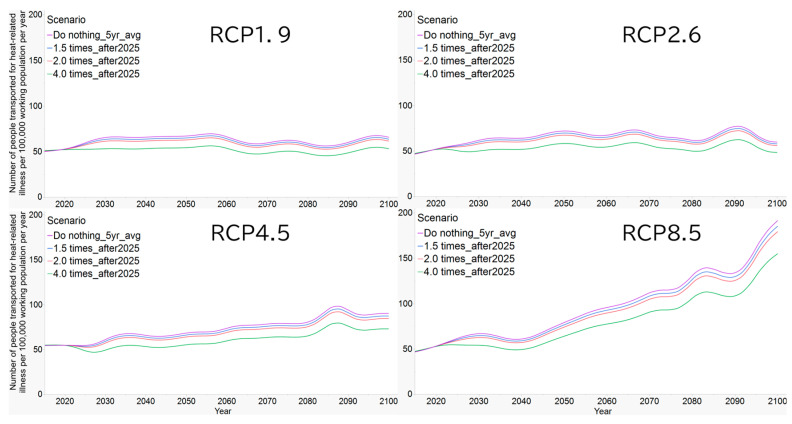
Long-term projections of heat-related ambulance transports and relative risk reduction under behavioral adaptation scenarios in Shiga Prefecture. Using MIROC6 for Otsu City, Shiga Prefecture, we conducted long-term projections of heat-related ambulance transports under each RCP scenario. We additionally present projected results under scenarios in which all four preventive behaviors—regular hydration, use of an air-cooling vest, workers checking their health condition before work, and recognition of warning alerts—are implemented. Lines represent smoothed projections (5-year moving average). We assumed that increased adherence to preventive behaviors was achieved in 2025. The scenarios labeled 1.5 times, 2.0 times, and 4.0 times correspond to relative increases of 50%, 100%, and 300% in the implementation rate of preventive behaviors, resulting in absolute coverages of 34%, 45%, and 91%, respectively. MIROC6, Model for Interdisciplinary Research on Climate 6; RCP, representative concentration pathway.

Figure 2 shows the long-term projection of heat-related ambulance transports through the year 2100 using MIROC6, stratified by the implementation coverage of preventive behaviors. Across all RCPs, the long-term increasing trend was not altered, and the projected number of ambulance transports exceeded the median of the baseline period (2018–2024). Even under the most intensive scenario (91% coverage), the population-level relative risk remained 0.81.

## 4. Discussion

In the present study, we projected the long-term future number of ambulance transports due to heat-related illness in the working population, using the daily maximum WBGT, and we evaluated the possible effectiveness of adaptation policies by actively increasing the coverage of (i) regular hydration, (ii) use of an air-cooling vest, (iii) workers checking their own health condition before work, and (iv) recognizing warning signs. Even when these four possible countermeasures were practiced 50% or 100% more frequently than currently, the expected relative risk reductions were 3.2% and 6.3%, respectively, indicating small effects on the future trend of heat-related illness. Although long-term projections regarding heatstroke using the WBGT are common worldwide [11,26], the present study is the first to assess this risk in the working population, evaluating possible countermeasures that are in line with recent changes in policies mandating prevention in occupational settings.

We highlight two take-home messages from the present study. First, the relative risk reductions as a result of possible behavioral interventions were limited. Even so, hydration and awareness about the risk of heat exposure as well as about warning signs constitute the core component of heat-related illness prevention in working populations. Although four preventive behaviors were consistently associated with lower risk of heat-related illness episodes in our survey, the expected population-level risk reduction by increasing the coverage rate of implementing all four behaviors was limited, largely because the effect size (i.e., preventive impact) was limited; consequently, the PAF of preventive behaviors was approximately 20%, implying that more than 80% of cases would not be prevented only by adopting preventive behaviors. Nevertheless, efforts to reduce preventable heat exposure will remain critical [27,28,29,30]. Additionally, if working conditions that involve exposure to heat continue to threaten worker safety, a fundamental overhaul of occupational management (e.g., drastic shift in working time to earlier morning, restricting or prohibiting work with excessive heat exposure) should be considered [31,32,33,34]. Heat stress is an invisible occupational hazard, calling for protection via work management systems, including legal restrictions [35].

The second take-home message is that even with a 100% increase in the implementation rate of behavioral interventions, the relative risk reduction was as low as 6.3%; thus, behavioral interventions may be insufficient as an adaptation strategy or policy. Considering that stronger interventions are unlikely to be readily accepted at work sites, relying on adaptation policies may not be the best approach to address the issue of climate change. Mitigating climate change itself is an essential requirement to ensure safe occupational activities.

An important technical contribution of the present study is the connection of our observational study results with long-term projection models of heat-related illness, using relative risk reduction, by varying the implementation coverage of behavioral interventions. We projected future heat-related ambulance transports among the working population and evaluated the expected population impact of feasible behavioral adaptation measures. A similar approach, but using the PAF, has been followed in older populations [23]. In the present study, we applied a multivariate model to bridge the gap between observational data and projections in the working population, showing that the maximally expected effect of adaptation policies may be 19% at most, which is very limited. As a side note in relation to the survey, interpretation of behavioral effect sizes requires caution. First, the survey was cross-sectional; therefore, estimated effect sizes represent associations rather than proven causal effects. Reverse causation is possible; individuals with prior heat-related illness or perceived higher risk may be more likely to adopt protective behaviors (e.g., monitoring and awareness). Second, linking survey-derived effect sizes to long-term climate projections assumes stability of effects over time; in practice, workplace technology, regulations, acclimatization, and baseline behaviors may change, altering future effectiveness.

Four technical limitations of this study must be discussed. First, the effect size in this study relied on cross-sectional observational data, and unobserved confounders could not be eliminated. However, rather than precisely estimating the causal impact, the role of the effect size was to show that the impact in reducing the future number of heat-related ambulance transports was limited. Thus, interpreting our results as tentatively quantitative findings is appropriate. Second, our WBGT-based projection model used daily maximum WBGT as a summary of heat exposure. This does not capture within-day exposure variations, short-term heat spikes, work–rest cycles, or individual acclimatization, which are particularly relevant in occupational settings. Future studies incorporating high-resolution exposure data, information on working hours, and workplace countermeasure implementation would improve precision. Third, the policy scenarios (50%, 100%, and 300% increases) are illustrative and intended to quantify potential upper bounds under strong assumptions. Although our results suggest that improving behavioral coverage alone may not sufficiently offset projected increases, system-level measures that reduce occupational heat exposure (e.g., work scheduling, engineering controls, and enforceable exposure limits) are plausible complementary strategies. However, these were not directly tested in our data and should thus be framed as policy considerations rather than empirically estimated effects. Moreover, various working conditions (e.g., industrial structure, ratio of white colors [reflect heat] to blue colors [absorb heat], the proportion of time spent outdoors, technical innovations in the working environment) are fixed in the present study; thus, the present study did not account for possible drastic changes in heat exposure. These features could directly impact the occupational risk of heat exposure and sustainability of working conditions [31,32,36]. Fourth, generalizability of the findings is limited. Shiga Prefecture provides an appropriate setting for modeling, but industrial structures, climate conditions, and outdoor work prevalence vary across regions. Although our findings may be directly applicable to Japanese prefectures that share similar industrial and climatological characteristics, this may not be the case globally.

## 5. Conclusions

The present study demonstrates that the number of heat-related ambulance transports is expected to increase, and that the relative risk reduction owing to behavioral interventions is likely limited. A fundamental overhaul of working rules, restrictions, and the environment (e.g., shift in working hours to earlier morning) should be considered as possible adaptation measures. Importantly, mitigation of climate change is vital.

## Figures and Tables

**Table 1:** Implementation rates of preventive behaviors by episode of heat-related illness (n = 1650).

Variables	With Heat-Related Illness Episode	No Episode
Age	45.5 ± 15.0	45.5 ± 15.0
Sex (male)	275 (50.0)	550 (50.0)
Single	225 (40.9)	489 (44.4)
Having at least one child	308 (56.0)	528 (48.0)
Having exercise habits	297 (54.0)	379 (34.4)
Regularly eating breakfast	387 (70.3)	857 (77.9)
Recognition of WBGT before work	192 (34.9)	214 (19.4)
Recognition of warning alert	280 (50.9)	621 (56.4)
Aware of the need for prevention	336 (61.0)	706 (64.1)
Monitoring WBGT	190 (34.5)	296 (26.9)
Air-cooling vest	265 (48.1)	648 (58.9)
Break time during work	281 (51.0)	530 (48.1)
Checking health conditions	202 (36.7)	469 (42.6)
Regular hydration	373 (67.8)	858 (78.0)

Age values are mean ± standard deviation (years); all other values are numbers (%). For binary variables, the proportion of those responding “yes” is shown (percentages do not sum to 100% across rows). WBGT, wet-bulb globe temperature.

**Table 2:** Univariate analysis with the presence of a heat-related illness episode as the dependent variable (crude odds ratios).

Variables	Odds Ratio (95% CI)	*p*-Value
Age	1.0 (1.0–1.0)	0.9425
Sex (male)	1.0 (0.8–1.2)	1.000
Single	0.9 (0.7–1.1)	0.1876
Having at least one child	1.4 (1.1–1.7)	0.0024
Having exercise habits	2.2 (1.8–2.8)	<0.0001
Regularly eating breakfast	0.7 (0.5–0.8)	0.0010
Recognition of WBGT before work	2.2 (1.8–2.8)	<0.0001
Recognition of warning alert	0.8 (0.7–1.0)	0.0359
Aware of the need for prevention	0.9 (0.7–1.1)	0.2337
Monitoring WBGT	1.4 (1.1–1.8)	0.0016
Air-cooling vest	0.6 (0.5–0.8)	<0.0001
Break time during work	1.1 (0.9–1.4)	0.2730
Checking health conditions	0.8 (0.6–1.0)	0.0200
Regular hydration	0.6 (0.5–0.7)	<0.0001

WBGT, wet-bulb globe temperature; CI, confidence interval.

**Table 3:** Effect sizes of preventive behaviors to reduce the risk of heat-related illness.

Preventive Behavior	Adjusted OR (95% CI)	
	Propensity Score Matched	IPTW
Regular hydrations	0.7 (0.5, 0.9)	0.6 (0.5, 0.7)
Air-cooling vest	0.7 (0.6, 0.9)	0.7 (0.6, 0.9)
Recognition of warning alert	0.7 (0.5, 0.9)	0.7 (0.6, 0.9)
Checking health condition	0.8 (0.7, 1.0)	0.8 (0.7, 1.0)

Propensity score matching included age, sex, presence of children, working environment, and underlying comorbidity as variables to be adjusted. IPTW, inverse probability of treatment weighting method; OR, odds ratio; CI, confidence interval.

**Table 4:** Relative reduction in heat-related illness risk when the coverage of preventive behaviors increased by 50%, 100%, or 300% (relative increases).

Policy Scenario (Relative Increase from Baseline)	Coverage Implementing All Four Behaviors (1 − w′)	$i_{v}\left(t\right)/i_{r}\left(t\right)$ (Cases Under Policy/Baseline)	Relative Risk Reduction in Heat-Related Ambulance Transports at the Population Level [95% CI]
50% increase (×1.5)	34%	0.968	3.2% [0.6, 5.4]
100% increase (×2.0)	45%	0.937	6.3% [1.2, 10.8]
300% increase (×4.0)	91%	0.810	19.0% [3.5, 32.3]

CI, confidence interval.

## Data Availability

The original data presented in the study are openly available as Supplementary Materials.

## Supplementary Materials

The following supporting information can be downloaded at: https://www.mdpi.com/article/10.3390/epidemiologia7030060/s1, Tables S1–S4: Modeled meteorological variables from 2015–2100, Table S5: Daily number of ambulance transports due to heat-related illness during 2018–2024.

## Abbreviations

The following abbreviations are used in this manuscript:

CI	confidence interval
MIROC	Model for Interdisciplinary Research on Climate
RCP	representative concentration pathway
WBGT	wet-bulb globe temperature

## References

[1] Bouchama A., Knochel J.P. (2002). Heat stroke. N. Engl. J. Med..

[2] Epstein Y., Yanovich R. (2019). Heatstroke. N. Engl. J. Med..

[3] Watts N., Amann M., Arnell N., Ayeb-Karlsson S., Belesova K., Boykoff M., Byass P., Cai W., Campbell-Lendrum D., Capstick S. (2019). The 2019 report of The Lancet Countdown on health and climate change: Ensuring that the health of a child born today is not defined by a changing climate. Lancet.

[4] Kjellstrom T., Holmer I., Lemke B. (2009). Workplace heat stress, health and productivity—An increasing challenge for low and middle-income countries during climate change. Glob. Health Action.

[5] Vicedo-Cabrera A.M., Scovronick N., Sera F., Royé D., Schneider R., Tobias A., Astrom C., Guo Y., Honda Y., Hondula D.M. (2021). The burden of heat-related mortality attributable to recent human-induced climate change. Nat. Clim. Change.

[6] Pascal M., Laaidi K., Ledrans M., Baffert E., Caserio-Schönemann C., Le Tertre A., Manach J., Medina S., Rudant J., Empereur-Bissonnet P. (2006). France’s heat health watch warning system. Int. J. Biometeorol..

[7] Fouillet A., Rey G., Wagner V., Laaidi K., Empereur-Bissonnet P., Le Tertre A., Frayssinet P., Bessemoulin P., Laurent F., De Crouy-Chanel P. (2008). Has the impact of heat waves on mortality changed in France since the European heat wave of summer 2003? A study of the 2006 heat wave. Int. J. Epidemiol..

[8] Kovats R.S., Kristie L.E. (2006). Heatwaves and public health in Europe. Eur. J. Public Health.

[9] Budd G.M. (2008). Wet-bulb globe temperature (WBGT)—Its history and its limitations. J. Sci. Med. Sport.

[10] Ueno S., Hayano D., Noguchi E., Aruga T. (2021). Investigating age and regional effects on the relation between the incidence of heat-related ambulance transport and daily maximum temperature or WBGT. Environ. Health Prev. Med..

[11] Fujimoto M., Nishiura H. (2022). Baseline scenarios of heat-related ambulance transportations under climate change in Tokyo, Japan. PeerJ.

[12] Oyama T., Takakura J., Ishizaki N.N., Oka K., Honda Y., Masago Y., Hijioka Y. (2025). Nationwide high-resolution heat risk projections and intervention cost analysis for the elderly in Japan under climate and demographic changes. Environ. Res..

[13] Tustin A.W., Lamson G.E., Jacklitsch B.L., Thomas R.J., Arbury S.B., Cannon D.L., Gonzales R.G., Hodgson M.J. (2018). Evaluation of Occupational Exposure Limits for Heat Stress in Outdoor Workers—United States, 2011–2016. Morb. Mortal Wkly. Rep..

[14] Ministry of Health, Labour and Welfare, Japan Strengthening Countermeasures Against Heat-Related Illness at Work. https://www.mhlw.go.jp/content/001476821.pdf.

[15] Fire and Disaster Management Agency (FDMA) (2025). Data on Emergency Transport Personnel Among Working Age Group Due to Heatstroke. https://www.fdma.go.jp/disaster/heatstroke/post4.html.

[16] Ministry of the Environment, Government of Japan (MOE) Heat Illness Prevention Information. Heat Stress Index: WBGT. https://www.wbgt.env.go.jp/record_data.php?region=07&prefecture=60&point=60216.

[17] Japan Meteorological Agency, Government of Japan (JMA). https://www.data.jma.go.jp/stats/etrn/index.php.

[18] National Institute for Environmental Studies Modeled Climatological Data. https://adaptation-platform.nies.go.jp/data/jma-obs/index.html.

[19] Tatebe H., Ogura T., Nitta T., Komuro Y., Ogochi K., Takemura T., Sudo K., Sekiguchi M., Abe M., Saito F. (2019). Description and basic evaluation of simulated mean state, internal variability, and climate sensitivity in MIROC6. Geosci. Model Dev..

[20] Yukimoto S., Kawai H., Koshiro T., Oshima N., Yoshida K., Urakawa S., Tsujino H., Deushi M., Tanaka T., Hosaka M. (2019). The Meteorological Research Institute Earth System Model Version 2.0, MRI-ESM2.0: Description and Basic Evaluation of the Physical Component. J. Meteorol. Soc. Jpn..

[21] Boucher O., Servonnat J., Albright A.L., Aumont O., Balkanski Y., Bastrikov V., Bekki S., Bonnet R., Bony S., Bopp L. (2020). Presentation and Evaluation of the IPSL-CM6A-LR Climate Model. J. Adv. Model Earth Syst..

[22] Ono M., Tonouchi M. (2014). Estimation of wet-bulb globe temperature using generally measured meteorological indices. Jpn. J. Biometeorol..

[23] Shiga Prefecture Past Population in Shiga Prefecture. https://www.pref.shiga.lg.jp/kensei/tokei/jinkou/maitsuki/335781.html.

[24] Climate Change Adaptation Information Platform. Future Population in Shiga Prefecture. https://adaptation-platform.nies.go.jp/data/socioeconomic/index.html.

[25] Fujimoto M., Hayashi K., Nishiura H. (2023). Possible adaptation measures for climate change in preventing heatstroke among older adults in Japan. Front. Public Health.

[26] Oka K., Phung V.L.H., He J., Honda Y., Hashizume M., Hijioka Y. (2025). Future heatstroke mortality in Japan: Impacts of climate, demographic changes, and long-term heat adaptation. Environ. Res..

[27] Bell M.L., Gasparrini A., Benjamin G.C. (2024). Climate change, extreme heat, and health. N. Engl. J. Med..

[28] Sorensen C., Hess J. (2022). Treatment and prevention of heat-related illness. N. Engl. J. Med..

[29] O’Connor F.G. (2025). Heat-related illnesses. Ann. Intern. Med..

[30] Guallar E., Bravo P.E., Ferrari V.A. (2024). Feeling the heat: Cardiovascular consequences of heat exposure under controlled experimental conditions. Ann. Intern. Med..

[31] Flouris A.D., Dinas P.C., Ioannou L.G., Nybo L., Havenith G., Kenny G.P., Kjellstrom T. (2018). Workers’ health and productivity under occupational heat strain: A systematic review and meta-analysis. Lancet Planet. Health.

[32] Andrews O., Le Quéré C., Kjellstrom T., Lemke B., Haines A. (2018). Implications for workability and survivability in populations exposed to extreme heat under climate change: A modelling study. Lancet Planet. Health.

[33] Zhao M., Chen Y., Shang J., Zhang S., Lu B., Miao Y., Lei M., Li R., Cai W., Zhang C. (2025). Potential of shifting work hours for reducing heat-related loss and regional disparities in China: A modelling analysis. Lancet Planet. Health.

[34] Dasgupta S., van Maanen N., Gosling S.N., Piontek F., Otto C., Schleussner C.F. (2021). Effects of climate change on combined labour productivity and supply: An empirical, multi-model study. Lancet Planet. Health.

[35] Mahase E. (2024). Heat stress: Billions of people are at risk from “invisible killer”, UN warns. BMJ.

[36] Meade R.D., González-Casabianca F., Rawal R., Venugopal V., Isaac T., Shaikh A., Wiskel T., Asrani S., Gadhvi D., Huybers P.J. (2025). Responding to rising heat in workplaces and homes of low income workers. BMJ.

